# What are the baseline measurements for physeal plate widths in healthy, uninjured children?

**DOI:** 10.1186/2036-7902-4-S1-A17

**Published:** 2012-12-18

**Authors:** Lorraine Ng, JA Martin, RE Lewiss

**Affiliations:** 1 St. Luke’s Roosevelt Hospital Center, Department of Emergency Medicine, Division of Emergency Ultrasound, New York, NY, USA

## Background

There is limited data on the sonographic evaluation of normative physeal plate measurements in healthy, uninjured children.

## Objectives

To determine the baseline measurements for physeal plate widths in healthy, uninjured children.

## Methods

This is a prospective observational study of a convenience sample of healthy patients between ages 0 and 12 years presenting to the pediatric emergency department. A focused ultrasound of the distal tibia, fibula, radius and ulna were performed bilaterally (8 total). Measurements were taken at the physeal plates in the longitudinal plane at the widest distance. The degree of variance of physeal plate widths within an individual and the average values of physeal plates for each bone were calculated.

## Results

A total of 60 patients were enrolled in this study. The mean age of enrolled patients was 6 years 3 months, 38% of who were female. Mean physeal plate diameters for the averaged measurement of each bone were: tibia 0.33 cm (95% CI 0.29 – 0.36), fibula 0.31 cm (95% CI 0.28 – 0.34), radius 0.27 cm (95% CI 0.24 – 0.30) and ulna 0.32 cm (95% CI 0.27 – 0.36). Mean values for the absolute difference in physeal plate diameters were: tibia 0.06 cm (95% CI 0.04 – 0.07), fibula 0.06 cm (95% CI 0.04 – 0.07), radius 0.05 cm (95% CI 0.04 – 0.07), and ulna 0.1 cm (95% CI 0.05 – 0.16). When measurements were stratified by age, the confidence intervals for each averaged measurement narrowed with increasing age while the absolute difference in physeal plate diameters remained consistent.

## Conclusion

This pilot study demonstrated that there was no statistically significant difference in physeal plate diameters between contralateral extremities and the degree of variation between contralateral extremities was minimal. Results of this study elucidate normative growth plate variance in healthy children and demonstrate that mean plate measurements and absolute differences are narrow. This study suggests that sonographic detection of significant disparities in physeal plate diameters of injured children may have the potential for earlier detection of Salter Harris injuries with subsequent appropriate referral and management.
